# The Chinese prescription lianhuaqingwen capsule exerts anti-influenza activity through the inhibition of viral propagation and impacts immune function

**DOI:** 10.1186/s12906-017-1585-7

**Published:** 2017-02-24

**Authors:** Yuewen Ding, Lijuan Zeng, Runfeng Li, Qiaoyan Chen, Beixian Zhou, Qiaolian Chen, Pui leng Cheng, Wang Yutao, Jingping Zheng, Zifeng Yang, Fengxue Zhang

**Affiliations:** 1 Guangzhou Institute of Respiratory Disease, State Key Laboratory of Respiratory Diseases, National Center for clinical research, The First Affiliated Hospital, Guangzhou Medical University, 1 Kangda Road, Guangzhou, 510230 China; 2 Institute of Tropical Medicine, Guangzhou University of Chinese Medicine, 12 Airport Road, Guangzhou, 510405 China; 3 Otolaryngological Department, Guangdong Provincial Hospital of Chinese Medicine, The Second Clinical College of Guangzhou University of Chinese Medicine, Guangzhou, China; 4 Faculty of Chinese Medicine, Macau University of Science and Technology, Avenida da Universidade, Taipa, Macau SAR 999078 China; 5 Science department, University of British Columbia, 2329 West Mall, Vancouver, BC V6T 1Z4 Canada

**Keywords:** Antiviral, Lianhuaqingwen capsule, Influenza virus, Immuno-regulation

## Abstract

**Background:**

Lianhuaqingwen Capsule (LH-C) is a traditional Chinese medicine (TCM) formula used to treat respiratory tract infectious diseases in Chinese. The aim of this study was to determine the antiviral activity of LH-C and its immunomodulatory effects on viral infection.

**Method:**

The in vitro cytotoxicity and antiviral activity of LH-C was determined by MTT and Plaque reduction assays. Time course study under single-cycle virus growth conditions were used to determine which stage of viral replication was blocked. The effect of LH-C on the nuclear export of the viral nucleoprotein was examined using an indirect immunofluorescence assay. The regulation to different signaling transduction events and cytokine/chemokine expression of LH-C was evaluated using Western blotting and real-time RT-PCR. After virus inoculation, BALB/c mice were administered with LH-C of different concentrations for 5 days. Body-weight, viral titers and lung pathology of the mice were measured, the level of inflammatory cytokines were also examined using real-time RT-PCR.

**Results:**

LH-C inhibited the proliferation of influenza viruses of various strain in vitro, with the 50% inhibitory concentration (IC_50_) ranging from 0.35 to 2 mg/mL. LH-C blocked the early stages (0–2 h) of virus infection, it also suppressed virus-induced NF-kB activation and alleviated virus-induced gene expression of IL-6, IL-8, TNF-a, IP-10, and MCP-1 in a dose-dependent manner. LH-C treatment efficiently impaired the nuclear export of the viral RNP. A decrease of the viral titers in the lungs of mice were observed in groups administered with LH-C. The level of inflammatory cytokines were also decreased in the early stages of infection.

**Conclusions:**

LH-C, as a TCM prescription, exerts broad-spectrum effects on a series of influenza viruses, including the newly emerged H7N9, and particularly regulates the immune response of virus infection. Thus, LH-C might be a promising option for treating influenza virus infection.

**Electronic supplementary material:**

The online version of this article (doi:10.1186/s12906-017-1585-7) contains supplementary material, which is available to authorized users.

## Background

Influenza virus, as a common respiratory pathogen, causes seasonal epidemics and occasional severe worldwide pandemics. The most recent event including the 2009 H1N1 pandemic (“swine flu”), and the 2013 H7N9 virus outbreak in China, which led to significant morbidity and mortality [1, 2].

Human influenza virus infections primarily affect the upper respiratory tract, resulting in clinical symptoms, including cough, fever, sore throat, rhinorrhea and congestion, occasionally followed by lower respiratory diseases like pneumonia [3, 4]. It has been reported that pneumonia causes approximately 80% mortality via influenza virus infection [5]. Upon pathogen infection of the respiratory tract, the host immune system is activated to resist and clear the infection. Airway epithelium cells and alveolar macrophages release multiple pro-inflammatory cytokines and chemokines, such as tumor necrosis factor (TNF-α), interleukin-6 (IL-6), interferon (IFN), and other chemokines, including IL-8, monocyte chemoattractant protein-1 (MCP-1), and macrophage inflammatory protein (MIP). This release results in the attraction and activation of additional inflammatory cells, including macrophages and neutrophils, into the lungs, initiating the innate immune system that is crucial for the clearance and resolution of viral particles [6, 7]. Factors implicated in severe influenza include robust cytokine production, otherwise known as the “Cytokine storm”. This effect has been considered one of the major contributors to the lethal disease caused by the 1918 pandemic strain and H5N1 viruses [8, 9].

Under physiological conditions, anti-inflammatory cytokines regulate the response of inflammation and attainment of equilibrium. However, the double-sided functions of cytokines could either be beneficial or detrimental to hosts. Under pathological conditions in which the balance is disrupted, pro-inflammatory responses may spiral out of control and excessive pro-inflammatory cytokines and inflammatory immune cells may contribute to additional tissue damage and inflammation [10, 11].

Vaccination is the most effective way to prevent influenza infection now. However, the high genetic variability of the virus renders the protection incomplete. In cases of a newly emerging strain, vaccination is only available a few months after the first appearance, leaving the population vulnerable during the crucial early phases of the pandemic [4]. Currently, two classes of antivirals are used as anti-influenza drugs: amantadine derivatives that blocking the virus-specific M2-ion channel and two neuraminidase (NA) inhibitors: oseltamivir (Tamiflu) and zanamivir (Relenza), both of which are approved by the FDA [12, 13]. Laninamivir was approved for the treatment of influenza in Japan in 2010. These drugs interfere with the activity of viral neuraminidase. In addition, the nucleoside analogues ribavirin and favipiravir (T-705) exhibit a suppressive effect against almost all RNA based human viruses [13]. However, resistant viruses against these prophylactic agents have emerged in recent years. Amantadine resistance has been detected in human and avian H5N1 strains, and an increasing number of clinical strains have been confirmed as resistant NA inhibitors, including oseltamivir and zanamivir [12, 14]. Additionally, all of these therapies are aimed at inhibiting virus propagation and spread; thus, the inflammation resulting from infection and the disease remain untreated. Because the severe outcome of influenza virus infection is associated with the aberrant production of inflammatory cytokines, maintaining the immune system in an appropriately robust condition may be detrimental for the prevention of the severe symptoms of influenza [15, 16].

LH-C composed of 13 herbs was extended from two TCM prescriptions: Maxing Shigan Tang and Yinqiao San. Maxing Shigan Tang was originally described in a classical Chinese book *Shanghan Lun* of Han Dynasty for the treatments of febrile diseases, it has been prescribed in treating bronchitis, pneumonia and early stage of measles [17]. Yinqiao San from the TCM monograph Wenbing Tiaobian of Qing Dynasty was mainly used for the treatment of “Warm disease” characterized by fever, thirst and headache. LH-C has been used in treating regular seasonal influenza for decades. Recently, A randomized controlled trial for the comparison of LH-C with oseltamivir in therapeutic effects on patients with mild H1N1 infection demonstrated that LH-C has a significant effect on the alleviation of fever, cough, sore throat and fatigue, it also showed comparative therapeutic effectiveness in reduction of illness duration and viral shedding duration [18, 19].

In the present study, we attempted to elucidate the mechanisms of LH-C anti-influenza activity, we examined the effect of LH-C on different influenza virus strains, and further addressed the impact of LH-C on the cell line and BALB/c mice, with particular focus on its anti-inflammation potential.

## Methods

### Reagent preparation

LH-C (Lot No. B1502001) was provided by Shijiazhuang Yiling Pharmaceutical Co., Ltd. (Shijiazhuang, China). The raw material of LH-C is black powder, comprising 13 ingredients as shown in Table 1. LH-C was dissolved in DMSO to 500 mg/mL and stored at −20 °C prior to use. Serum-free medium or saline was used as the dilution buffer in the follow-up experiments.

**Table 1:** Composition of LH-C

Plant	Family	Weight	Used part
*Forsythia suspensa* (Thunb.) Vahl	Oleaceae	255 g	Fructus
*Ephedra sinica* Stapf	Ephedraceae	85 g	Stem
*Lonicera japonica* Thunb.	Caprifoliaceae	255 g	Flower bud
*Isatis indigotica* Fortune	Brassicaceae	255	Root
*Mentha haplocalyx* Briq.	Mentha	7.5 g	Menthol
*Dryopteris crassirhizoma* Nakai	Dryopteridaceae	255 g	Rhizoma
*Rhodiola rosea* L.	Crassulaceae	85 g	Rhizoma
Gypsum Fibrosum	–	255 g	–
*Pogostemon cablin* (Blanco) Benth.	Labiatae	85 g	Whole plant
*Rheum palmatum* L.	Polygonaceae	51 g	Rhizoma
*Houttuynia cordata* Thunb.	Saururaceae	255 g	Whole plant
*Glycyrrhiza uralensis* Fisch.	Leguminosae	85 g	Rhizoma
*Armeniaca sibirica* (L.) Lam.	Rosaceae	85 g	Seed

### Cells and viruses

Influenza virus A/PR/8/34 (H1N1), B/Lee/1940, A/Guangdong/GIRD02/09 (H1N1), A/Aichi/2/68 (H3N2), A/Hongkong/1/68(H3N2), A/Duck/Hongkong/Y280/97(H9N2), A/Duck/Guangdong/09 (H6N2), and A/Shanghai/01/2013(H7N9) were propagated in the allantoic cavity of chicken eggs. An oseltamivir-resistant variant of H1N1 influenza virus A/PR/8/34 (H1N1) (H274Y mut) and mouse-adapted influenza virus (A/PR/8/34, H1N1) was propagated in MDCK cells. The virus titers were determined based on a 50% tissue culture infectious dose (TCID_50_) assay. Madin-Darby canine kidney (MDCK) cells and A549 cells, a human alveolar type II-like epithelial cell line, were cultured in a monolayer in Minimum Essential Medium (MEM) or Dulbecco’s modified Eagle’s medium (DMEM) respectively, supplemented with 10% fetal bovine serum (FBS), penicillin (100 U/mL) and streptomycin (10 μg/mL), incubated at 37 °C under 5% CO_2_. In vitro experiments were conducted in a biosafety level 2 containment facility. All procedures involving live H7N9 viruses were conducted at a biosafety level 3 facility.

### Animals

Specific-pathogen-free BALB/c female mice weighing approximately 16 to 18 g were purchased from Guangdong Medical Laboratory Animal Center (Guangzhou, China). The animals were fed a standard laboratory diet and provided water ad libitum. The animal experiments were performed in accordance with the Guidelines of Guangdong Regulation for the Administration of Laboratory Animals.

### Cytotoxicity assay

MDCK cells were plated onto 96-well plates and cultured to reach 80–90% confluence at 37 °C under 5% CO_2_ for 24 h. The aspirated medium contained various concentrations of LH-C (0.625–20 mg/mL, 100 μL/well), and the cells were further incubated at 37 °C for 48 h. Approximately 20 μL of Methyl Thiazolyl Tetrazolium (MTT) at concentration of 5 mg/mL was added to each well, and the cells were further incubated at 37 °C for 4 h. The medium was subsequently removed, and formazan crystals were solubilized with dimethyl sulfoxide (DMSO) (100 μL/well). The absorbance was measured at 490 nm using a microplate reader [20]. The 50% toxic concentration (TC_50_) was calculated using the Reed-Muench analysis [21].

### Antiviral assay

The anti-influenza virus activity of LH-C was examined using cytopathogenic effect (CPE) and MTT assays [22]. Briefly, MDCK cells were seeded onto 96-well plates and infected with 100 TCID_50_/100 μl of influenza virus at 37 °C for 2 h. The medium was aspirated, and the cells were incubated with 100 μl of serum-free MEM containing 1.5 μg/ml of trypsin, antibiotics and various concentrations of LH-C (0.03125-2 mg/mL) at 37 °C under 5% CO_2_ for 2–3 days. LH-C was dissolved in DMSO and diluted in culture medium to obtain various final concentrations. The concentration of DMSO in each medium was less than 1%. Approximately 20 μL/well of MTT (5 mg/ml) was subsequently added into each well, and the cells were further incubated for 4 h at 37 °C in a CO_2_ incubator. The crystallized formazan in the plates was dissolved in DMSO (100 μL/well). The absorbance was measured at 490 nm using a computer-controlled microplate reader (Bio-Rad, Tokyo, Japan) [20].

### Plaque reduction assay

MDCK cells (5 × 10^5^ cells/well) were plated onto 12-well culture plates and incubated for 24 h. The cells were washed twice with phosphate-buffered saline (PBS) prior to incubation with viruses (including A/PR/8/34 (H1N1), A/Hongkong/1/68 (H3N2), oseltamivir-resistant viruses (H1N1) and A/Guangzhou/GIRD02/09(H1N1) diluted in serum-free MEM containing 1% penicillin and streptomycin for 2 h at 37 °C. After incubation, the cell monolayer was covered with overlay medium containing LH-C and further cultured at 34 °C under 5% CO_2_ for 72 h. Subsequently, the overlay medium was removed, and the cell monolayer was fixed with 10% formalin, stained with 1% crystal violet, and the plaques were counted [23].

### Time course assay

MDCK cells in 48-well plates were infected with virus A/PR/8/34 (H1N1) (MOI =0.1) for 2 h. After infection, the supernatant was removed, and the cells were rinsed twice with PBS. LH-C (2 mg/mL) was added to cells at 0, 2, 4, 6, 8 and 10 h after infection. The time of addition studies were conducted under single-cycle virus growth conditions. The supernatant was harvested at 12 h post infection, and the virus titers were determined in MDCK cells [24].

### Indirect immunofluorescence assay

MDCK cells were seeded onto 48-well plates (200 μL/well); when the cell culture reached 50% at 37 °C under 5% CO_2_, the virus A/PR/8/34(H1N1) (MOI =0.1) was infected for 2 h. After incubation, the supernatant was aspirated, the cells were washed twice with PBS, and LH-C (2 mg/mL) was subsequently added to cells, followed by incubation at 37 °C under 5% CO_2_. At 6 and 8 h post infection, the cells were fixed with 4% PFA in PBS for 30 min at 4 °C The cells were permeabilized with 0.5% Triton X-100 in PBS for 15 min at room temperature and blocked with 5% BSA in PBS for 20 min at 37 °C, adding anti-influenza A virus NP antibody overnight at 4 °C. After further washing, the cells were incubated with FITC-labeled goat anti-mouse IgG at 37 °C for 1 h. The nuclei were stained with DAPI (5 μg/mL), and fluorescence was visualized using a Zeiss Axiovert 135 fluorescence microscope.

### RNA extraction, reverse transcription, and real-time quantitative PCR (qRT-PCR)

The relative gene expression in A549 cells infected with A/Puerto Rico/8/34 H1N1 was analyzed using qRT-PCR. Total RNA was extracted with 1 ml of TRIzol^TM^ reagent (Invitrogen Life Technologies) and dissolved in RNase-free water. One microgram from each RNA extract was used to generate first-strand cDNA using the PrimeScript RT-PCR Kit (Takara Bio) using both oligo (dT) and random primers. qRT-PCR was performed using an ABI7500 system (Applied Biosystems) with the following conditions: 95 °C for 30 s, followed by 40 cycles of 95 °C for 5 s and 60 °C for 30 s. Forward and reverse primers in combination with FAM/TAMRA probes sequences for IL-6, IL-8, IP-10, TNF-α, MCP-1 and GADPH genes were previously published and listed in Additional file 1: Table S1. Relative gene expression levels were calculated as 2^-△△CT^ (Sym et al., [4]).

### Western blotting

A549 cells were inoculated into a culture flask containing DMEM/F12 (1:1) culture medium (HyClone, Thermo Scientific Inc.) supplemented with 10% (v/v) fetal bovine serum (FBS). After growing to over 80% confluence, the cells were seeded onto 6-well plates at a density of 2x10^5^ and subsequently infected with influenza A virus (PR8) (MOI = 0.1) in the absence or presence of different concentrations of LH-C. The cells were washed twice with cold PBS and subsequently lysed in commercial RIPA lysis buffer (Beyotime) containing Complete, Mini, EDTA-free protease inhibitor cocktail (Roche). Protein concentrations were determined using the BCA Protein Assay kit (Pierce) according to the manufacturer’s instructions. The proteins were separated using SDS-PAGE and subsequently transferred to PVDF membranes, followed by blocking for an hour at room temperature in 5% nonfat milk in TBST. Following incubation with antibodies against phospho Ser536-NF-kBp65, phospho Thr202/Tyr204-ERK, NF-kB, ERK, GAPDH (Cell Signaling) and a secondary HRP-conjugated antibody, the immunocomplexes were detected using enhanced chemiluminescence (ECL, Amersham).

### Mouse inoculation and anti-viral treatment

The mice were intranasally infected with 2 MLD_50_ of mouse-adapted A/PR/8/34 (H1N1) virus in a volume of 50 μL. Groups of mice were orally administered 1300 and 650 mg/kg/day of LH-C solution respectively. The control animals were treated with the solvent only. The drug was administered twice a day (at 12-h intervals) for 5 days.

### Sample collection, process and detection

One set of 14 mice was monitored for weight loss from 3 days before to 15 days post infection of the virus. A second set of animals was sacrificed at 4, 6, and 8 days after infection and the lung samples were harvested. Lung tissues from euthanized mice were extracted and homogenized in MEM containing antibiotics (0.1% penicillin-streptomycin). The obtained specimens were centrifuged at 12,000 rpm for 5 min at 4 °C, aliquoted and stored at −80 °C for further analysis. The lung homogenates were determined according to the virus titer using end-point titration in MDCK cells and real-time RT-PCR for mRNA expression as previously described.

### Histopathological analysis

The lungs were inflated with 10% formaldehyde solution. The tissues were processed for paraffin embedding and cut into 4-μm-thick sections. The tissue samples were subjected to standard hematoxylin and eosin staining.

### Statistical analysis

The data are expressed as the means ± S.E.M. Statistical differences between two groups were determined using Student’s *t* test. For multiple groups, one-way ANOVA analysis was used to compare the means. Statistical analyses were conducted using SAS 9.1. *P* < 0.05 was considered statistically significant. For survival studies, a log-rank (Mantel-Cox) test using GraphPad Prism (GraphPad 5.0 Software) was conducted.

## Results

### In vitro anti-influenza activity of LH-C against different influenza viruses

**Fig. 1:**
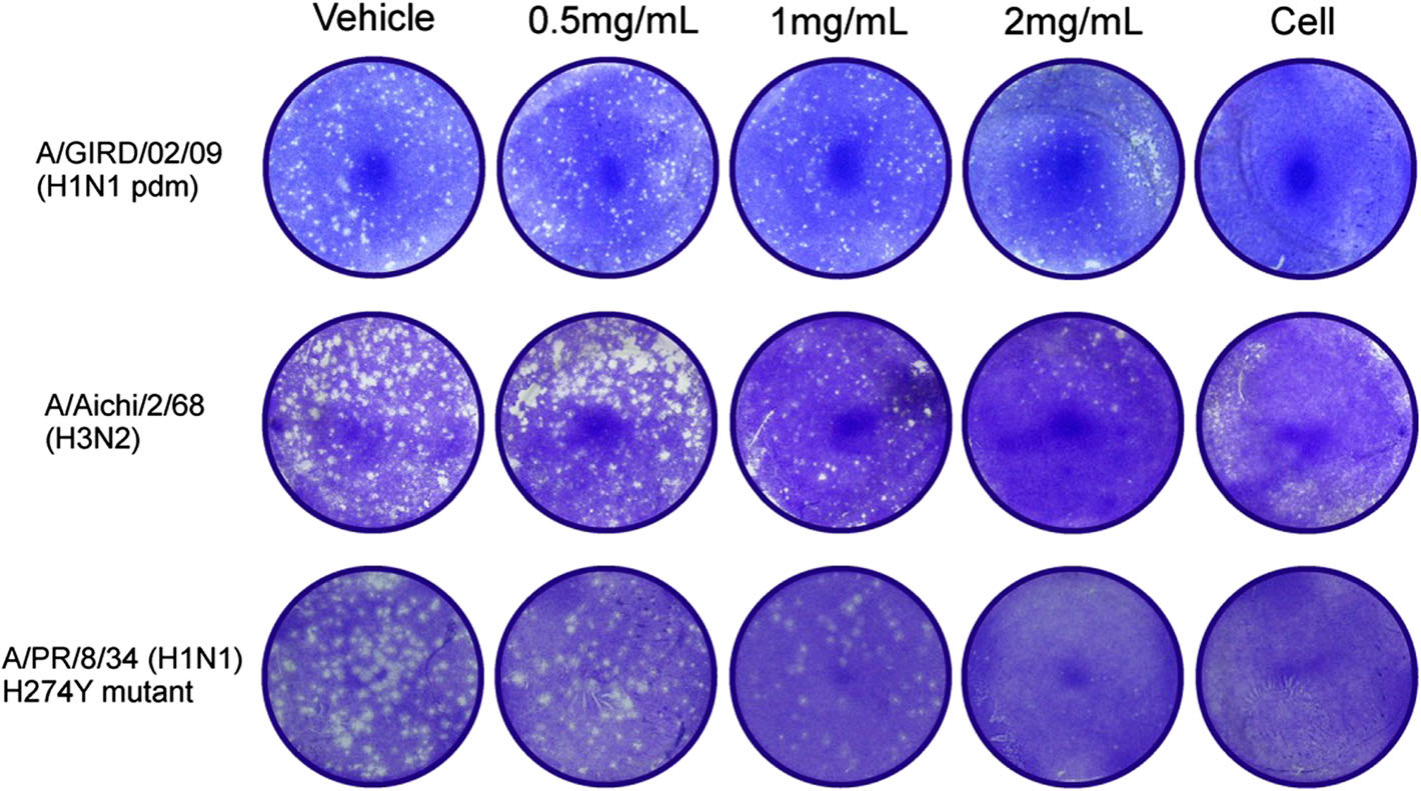
Plaque reduction assay of LH-C against influenza viruses. MDCK cells were infected with influenza viruses, including A/GIRD02/09 (H1N1), A/Aichi/2/68 (H3N2) and A/PR/8/34 (H1N1) (H274Y mut) at 0.01 MOI for 2 h at 34 °C. After viral adsorption, cell monolayer was covered with overlay medium containing LH-C in different concerntration. After incubation for 72 h, the cells were fixed and stained with crystal violet

In a first set of experiments we investigated whether LH-C exerts antiviral effect against influenza viruses infection in cultured cells. Therefore the cells were infected with virus and incubated for 72 h with LH-C at various concentrations. For infection, we used different virus strains, including the highly pathogenic avian influenza (HPAI) H7N9 influenza virus A/Shanghai/01/2013 (H7N9), swine-origin influenza A virus A/Duck/Guangdong/2009 (H6N2) and/Duck/Guangdong/Y280/92 (H9N2) and the influenza B virus prototype isolate B/Lee/1940. As shown in Table 2, LH-C exhibited inhibitory activities against several influenza viruses from different human isolates and avian influenza viruses, with IC_50_ ranging from 0.35 to 2 mg/mL, and a selective index (SI) ranging from 1.56 to 15.6. LH-C showed the best inhibitory effect in A/Aichi/2/68(H3N2) and A/Duck/Guangdong/Y280/92 (H9N2) (SI = 15.85 and 8.80, respectively). Interestingly, LH-C displayed no effect towards B/Lee/1940. The anti-influenza virus activities of LH-C against A/GIRD02/09 (H1N1), A/Aichi/2/68 (H3N2) and A/PR/8/34 (H1N1) (H274Y mut) were examined by plaque reduction assay (Fig. 1).

**Table 2:** Antiviral activity of LH-C against influenza viruses

Virus type and strain	LH-C	
	IC_50_ (mg/ml)^a^	SI
A/PR/8/34 (H1N1)	0.51	6.21
A/Guangzhou/GIRD02/2009 (H1N1)	0.71	4.46
A/Aichi/2/68 (H3N2)	0.2	15.85
B/Lee/1940	2	1.59
A/Duck/Guangdong/2009 (H6N2)	1	3.17
A/Duck/Guangdong/Y280/92 (H9N2)	0.42	8.80
A/Shanghai/01/2013 (H7N9)	0.85	3.68

The concentration required for TC_50_ of LH-C was 3.17 mg/ml to MDCK cells based on the MTT assay

^a^Mean of the results from three independent experiments

### LH-C treatments blocked the early stages of viral replication process

**Fig. 2:**
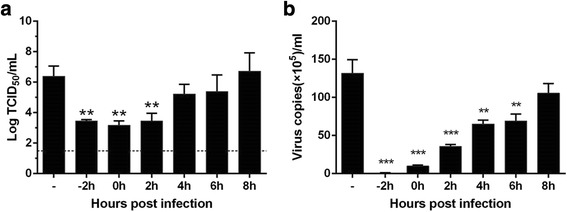
MDCK cells in 48-well plates were prepared and subsequently infected with virus A/PR/8/34 (H1N1) (MOI =0.01) for 2 h, after that LH-C (2 mg/mL) was added to the cells at −2, 0, 2, 4, 6 and 8 h after infection. The supernatants were collected and infectious titers were determined by MTT assay (**a**) and real time PCR assay (**b**). The data represent the means ± SD of 3 biological samples. The data are presented as the means ± SEM of three independent experiments. *P < 0.05, **P < 0.01, ***P < 0.001

To determine the step in the virus replicate cycle that is affected by LH-C, supernatants from cells that were treated with LH-C at different time points pre- and post-infection were analyzed for their content of progeny virus (Fig. 2a) and viral gene expression (Fig. 2b). Interestingly, most prominent reductions of virus titers were obtained in the early stages of viral replication (0–2 h). In the late stages of viral replication (4–8 h), LH-C displayed an insignificant effect. It suggested that the antiviral mechanism of LH-C involves the inhibition of early-stage influenza virus replication.

### LH-C inhibits nuclear export of virus nucleocapsid protein (RNP) in the infected A549 cells

**Fig. 3:**
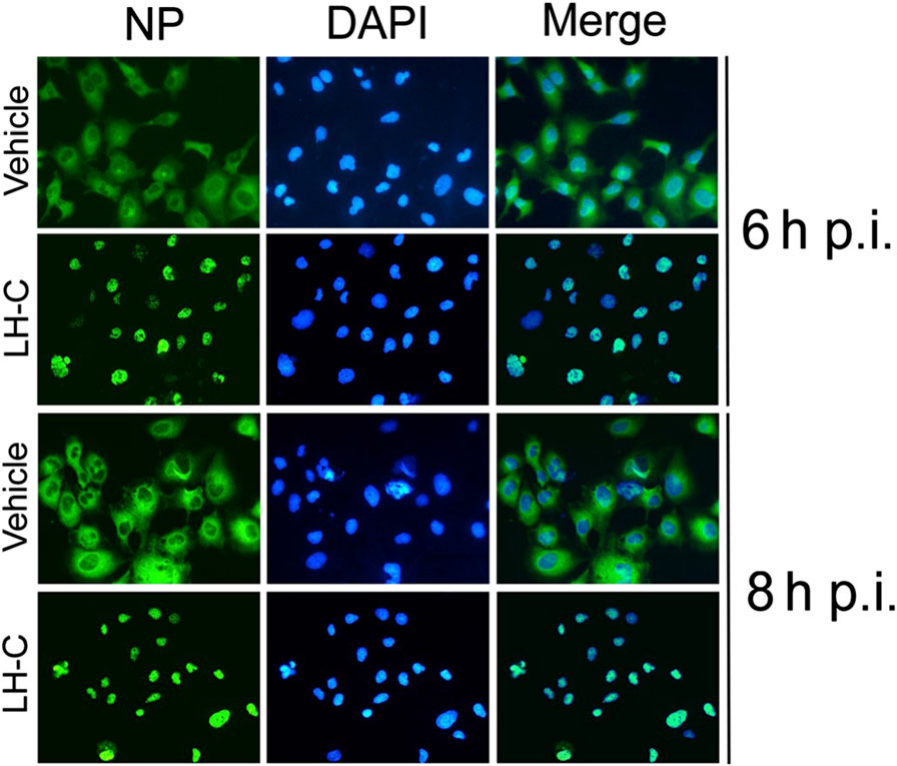
Indirect immunofluorescence microscopy. A549 cells were infected with A/PR/8/34 (H1N1) (MOI = 0.1) and treated with LH-C (2 mg/mL) or vehicle. After 6 and 8 h post infection, the cells were fixed for 30 min at 4 °C. Cell nuclei were stained with DAPI (blue) and viewed using a fluorescence microscope (Magnification 400×)

Earlier findings clearly indicated that the inhibition of NF-kB pathway resulted in efficient retention of influenza virus RNP complexes in the nucleus of infected cells without affecting accumulation of viral proteins [25]. Therefore, we investigated whether LH-C could block nuclear export of viral RNP in the infected A549 cells. As shown in Fig. 3, Immunofluorescence staining of the viral NP, which is one of the constituents of the RNP complexes reveals that the export of viral RNP were efficiently impaired in the presents of LH-C.

### LH-C inhibits influenza A virus-induced NF-κB activation in A549 cells

**Fig. 4:**
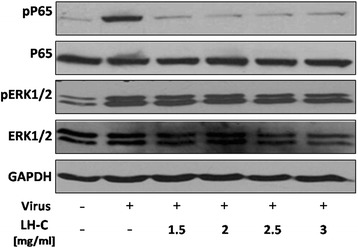
Effect of LH-C on influenza virus-induced signaling expression in A549 cells. A549 cells infected with A/PR/8/34(H1N1) (MOI = 0.1) were treated with 1.5–3 mg/ml LH-C for 24 h. Whole-cell lysates were prepared at the indicated time points, and an immunoblot analysis of the activity of NF-κB and MAPKs ERK1/2 was performed using phospho-specific and total expression antibodies

A variety of cellular signal pathways can be triggered in response to influenza A virus infection [26, 27]. The activation these signal pathways can be manipulated by viruses for efficient replication or the induction of host defense mechanisms against invading viruses [28]. Thus, we examined the effect of LH-C on different signaling transduction mechanisms after influenza A virus infection. Viral infection significantly increased the activation of NF-κB and Raf/MEK/ERK signaling in human alveolar epithelial (A549) cells (Fig. 4, Lane 1, 3). LH-C treatment suppressed influenza A virus-induced NF-kB activation but not the Raf/MEK/ERK cascade in virus-infected cells over a 24-h period (Fig. 4, Lane 3).

### LH-C inhibits influenza A virus-induced cytokine/chemokine expression in A549 cells

**Fig. 5:**
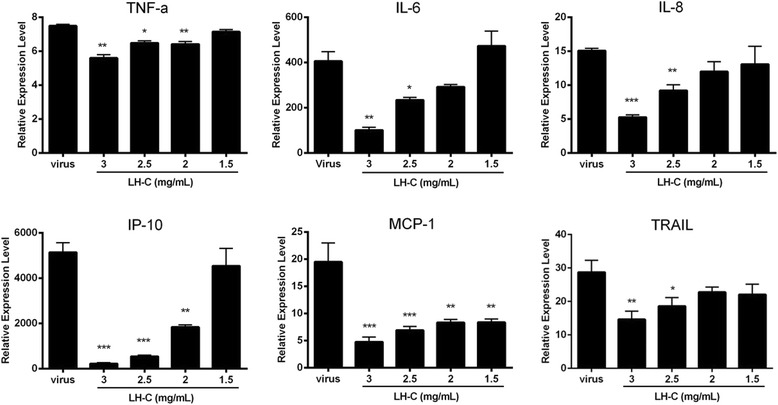
Effect of LH-C on influenza virus-induced cytokine/chemokine mRNA expression in A549 cells. A549 cells infected with A/PR/8/34(H1N1) (MOI = 0.1) were treated with 1.5–3 mg/ml LH-C for 24 h prior to extracting RAN. The cytokine/chemokine mRNA level of IL-6, IL-8, IP-10, TNF-α, and MCP-1 was analyzed using qRT-PCR. The data are presented as the means ± SEM of three independent experiments. *P < 0.05, **P < 0.01, ***P < 0.001

NF-κB pathway plays as a major regulator of cytokine and chemokine expression after influenza virus infection [28]. The overexpression of cytokines and chemokines induced through influenza virus infection depends on the NF-κB signaling pathway. A previous study showed that influenza A/PR/8 virus infection with specific NF-κB inhibitor treatment decreased the production of inflammatory cytokines, including IL-8 and IL-6 [29]. To examine the NF-kB inhibition effect of LH-C on virus-induced inflammatory responses, we used qRT-PCR to measure cytokine/chemokine expression in A549 cells infected with PR8 (MOI = 0.1) at 24 h p.i. As shown in Fig. 5, virus infection induced a robust increase in the gene expression of IL-6, IL-8, TNF-α, IP-10, MCP-1, whereas LH-C treatment exhibited a prominent inhibitory effect in a dose-dependent manner.

**Fig. 6:**
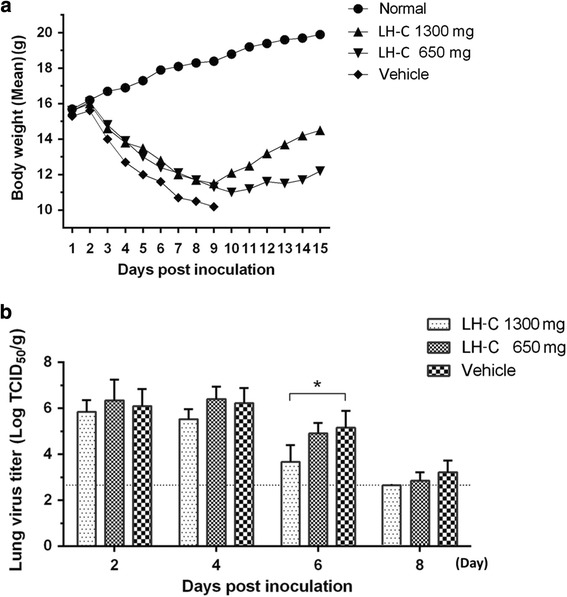
Administration of LH-C efficiently reduced influenza A virus replication in vivo. Three BALB/c mice per group were intranasally infected with 2 MLD_50_ of A/PR/8/34 (H1N1) virus. The mice were orally administered LH-C (650 or 1300 mg/day). **a** Mice were monitored for changes in body weight daily. **b** Influenza virus titers were detected in mice lungs at 2, 4, 6 and 8 days post inoculation. The data are presented as the means ± SEM of 3 mice per group. *P < 0.05, **P < 0.01, ***P < 0.001

### Administration of LH-C exhibits antiviral functions in vivo

To confirm the viral inhibitor properties of LH-C, groups of mice are inoculated with A/PR/8 (H1N1) virus in 2 MLD_50_ and subsequently administered LH-C and placebo, respectively. At 2, 4, 6 and 8 days post infection; the lungs were then collected from infected mice (3 mice per day) for the detection of the virus titer. As is shown in Fig. 6, A decrease (>2 log) of the viral titers in the lungs of mice by TCID_50_ was observed in groups administered LH-C (1300 mg/kg/day) compared with placebo at 6 and 8 days post challenge. Low-dosage LH-C treatment groups also presented reduced lung viral titers, although these values were not significantly different from those of the control group. These results suggested that LH-C has potential antiviral activity against influenza A viruses in mice.

### Administration of LH-C reduced the inflammatory response in the lungs of mice after influenza A virus infection

**Fig. 7:**
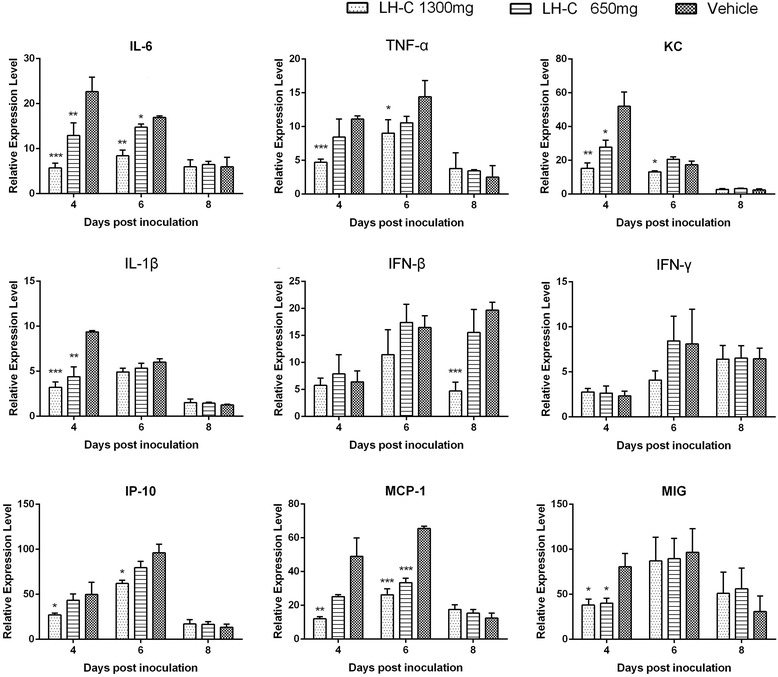
Inflammatory cytokines and chemokines were detected at the indicated days post infection. The cytokine/chemokine mRNA level was analyzed using qRT-PCR. The data are presented as the means ± SD.*P < 0.5, **P < 0.01, ***P < 0.001

To determine the properties of LH-C in modulating cytokine production during influenza virus infection, we examined the level of cytokines in the lungs of infected mice at 4, 6 and 8 days post infection. As shown in Fig. 7, the expression of pro-inflammatory cytokines (TNF-α and IL-6) and chemokines (KC and MCP-1) was strongly reduced in the presence of LH-C from 4 to 6 days post infection. The levels of IL-1β and IP-10 were also suppressed in a dose-dependent manner.

**Fig. 8:**
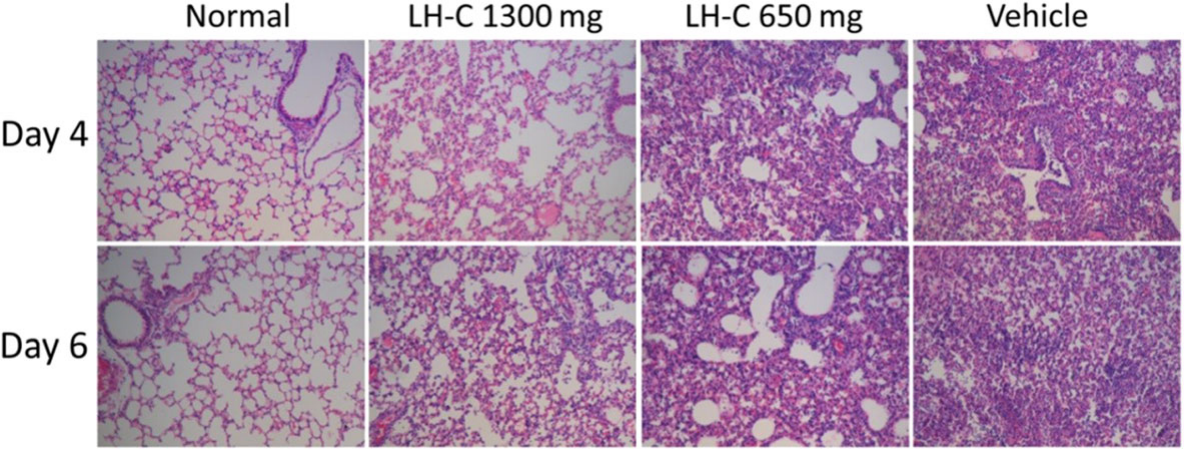
lung histology was examined at 4 and 6 days post inoculation. Sections of the lung tissues were visualized using hematoxylin and eosin (H&E) staining. (Magnification: 100×)

### Administration of LH-C reduced lung pathology induced by influenza infection

Lung histology was examined to determine whether LH-C treatment alleviated lung pathology resulting from influenza infection. As shown in Fig. 8, using hematoxylin and eosin (HE) staining, the lung tissues of infected animals showed considerable inflammation, with cell exudates in the lung parenchyma and small airways. Following LH-C treatment, the significant eradication of perivascular inflammation and fewer cell exudates were observed. Considering previous results showing viral loads following LH-C administration, these results suggested that LH-C treatment improved the lung pathology of influenza-infected mice.

## Discussion

LH-C has been widely used for more than 10 years in China. Previous studies have confirmed the curative effects of LH-C on several diseases, such as acute bronchitis, asthma, and COPD. A randomized controlled trial for the comparison of LH-C with oseltamivir in therapeutic effects on mild H1N1 infection demonstrated that LH-C has a comparative effect in viral clearance and performs even better in symptom relief [18].

However, the mechanism of LH-C action remains unclear. In this study, demonstrated that LH-C could inhibit different strains of influenza viruses, including HPAI A (H7N9) virus, the newly emerged A (H1N1) pdm09 virus and oseltamivir-resistant viruses (A/PR/8 H274Y). Treatment with LH-C following infection had an inhibitory effect on plaque reduction of the human or avian influenza viruses tested. Additionally, LH-C inhibited viral replication when added between 0 and 6 h, and particularly 0–2 h, after infection.

Similar to all other viral pathogens, influenza virus utilizes the host cellular machinery to support replication. NF-kB pathway plays an important role in the maintenance of host defense responses [30], independent studies have demonstrated that the pathway is critical for the efficient replication of influenza virus. The results of our experiments that LH-C could suppress A/PR/8/34 virus-induced phosphorylation of p65 in cells. Previous reports have demonstrated that viruses support NF-kB-dependent expression of proapoptotic factor, FasL and TRAIL, which activates caspases that subsequently regulate the nuclear export of the viral RNP complexes. Here, we demonstrated that LH-C could block the nuclear export of the viral RNP regardless of the virus-induced activation of Raf/MEK/ERK pathway, indicating that the antiviral effect of LH-C was predominantly via its NF-kB inhibiting activity to suppress viral RNP export and subsequent viral propagation. The NF-kB signaling pathway is not only involved in viral replication but is also the main regulator of cytokine and chemokine production in general and particularly during severe influenza infections [31–33]. We also demonstrated that the levels of cytokine/chemokine mRNA (including IL-6, 8, MCP-1 MIG, and IP-10) in infected cells were reduced in the presence of LH-C, indicating the regulatory activity of LH-C in an NF-kB-dependent manner. Blocking the NF-kB pathway as a potent strategy in influenza treatment has recently been considered, as this strategy will not only block virus propagation but also inhibit the development of related inflammation [25–29]. Previous studies have reported that NF-kB inhibitors show a considerable protective effect in mice against HPAI A virus infection [30], indicating the availability of NF-kB inhibitors for the treatment of HPAI virus.

Inflammatory cytokines and chemokines are produced during influenza virus infection. However, the multiple functions of cytokines could either be beneficial or detrimental to virus-infected hosts. To assess whether the antiviral and anti-inflammation properties of LH-C observed in cell culture would also be relevant in vivo compared with the placebo group, a notable pattern of regulation with cytokine was observed in LH-C-treated mice, particularly in NF-kB-dependent cytokines. We observed that the production of pro-inflammation TNF-α and IL-6 and the immunoregulatory IFN-β, MCP-1 and KC were significantly decreased later in infection (at 6 or 8 dpi) compared with non-treated mice, indicating the accelerated recovery from the immunity situation resulting from infection.

The adoptive concentrations (650 and 1300 mg/d) used in the in vivo study were based on the practical concentrations used in humans, though these concentrations were higher than conventional medicines, the results still showed no toxic side effects in mice, which is still in the range of the safety dose. In conclusion, LH-C might be a promising option as a new antiviral agent to fight IAV infections.

## Conclusions

LH-C, as a TCM prescription, has shown a broad spectrum of effects on a series of influenza viruses, including the newly emerged H7N9. LH-C exerts its anti-influenza activity by interfering with both viral and host reactions. Specifically, LH-C regulates the immune response of virus infection. Thus, LH-C might be a promising option in treating Influenza disease.

## Additional file

Additional file 1: Table S1. Primers and probes sequences. (DOC 73 kb)

## Abbreviations

CPE: Cytopathogenic effect
DMEM: Dulbecco’s modified Eagle’s medium
DMSO: Dimethyl sulfoxide
DMSO: Dimethyl sulfoxide
ERK: Extracellular signal-regulated kinase
FBS: Fetal bovine serum
HPAI: Highly pathogenic avian influenza
IC50: 50% inhibitory concentration
IFN: Interferon
IL-6: Interleukin-6
LH-C: Lianhuaqingwen capsule
MCP-1: Monocyte chemoattractant protein-1
MDCK: Madin-Darby canine kidney
MEM: Minimum Essential Medium
MIP: Macrophage inflammatory protein
MTT: Methyl Thiazolyl Tetrazolium
NA: Neuraminidase
PBS: Phosphate-buffered saline
qRT-PCR: Real-time quantitative PCR
SI: Selective index
TC50: 50% toxic concentration
TCID50: 50% tissue culture infectious dose
TCM: Traditional Chinese medicine
TNF-α: Tumor necrosis factor
