# Supplementary material for: The Chinese prescription lianhuaqingwen capsule exerts anti-influenza activity through the inhibition of viral propagation and impacts immune function
Source: BMC Complement Altern Med. 2017 Feb 24;17:130. doi: 10.1186/s12906-017-1585-7 (PMC5324200; doi:10.1186/s12906-017-1585-7)
Supplement: Additional file 1: Table S1. — Primers and probes sequences. (DOC 73 kb) [file 12906_2017_1585_MOESM1_ESM.doc]

**Table S1. Primers and probes sequences**

| Primer name | Sequences |
| --- | --- |
| IL-6 | (Forwardprimer5’-3’)ACAACCACGGCCTTCCCTACTT |
| (mouse) | (Reverseprimer5’-3')CACGATTTCCCAGAGAACATGTG |
|  | (Probe5’-3')5'FAM-TTCACAGAGGATACCACTCCCAACAGACCT-3'TAMRA |
|  |  |
| MCP-1 | (Forwardprimer5’-3’)CCACTCACCTGCTGCTACTCAT |
| (mouse) | (Reverseprimer5’-3')TGGTGATCCTCTTGTAGCTCTCC |
|  | (Probe5’-3')5'FAM-CACCAGCAAGATGATCCCAATGAGTAGGC-3'TAMRA |
|  |  |
| IL-1β | (Forwardprimer5’-3’)AACCTGCTGGTGTGTGACGTTC |
| (mouse) | (Reverseprimer5’-3')CAGCACGAGGCTTTTTTGTTGT |
|  | (Probe5’-3')5'FAM-TTAGACAGCTGCACTACAGGCTCCGAGATG-3'TAMRA |
|  |  |
| TNF-α | (Forwardprimer5’-3’)CATCTTCTCAAAATTCGAGTGACAA |
| (mouse) | (Reverseprimer5’-3')TGGGAGTAGACAAGGTACAACCC |
|  | (Probe5’-3')5'FAM-CACGTCGTAGCAAACCACCAAGTGGA-3'TAMRA |
|  |  |
| IFN-β | (Forwardprimer5’-3’)GCACTGGGTGGAATGAGACT |
| (mouse) | (Reverseprimer5’-3')TCCCACGTCAATCTTTCCTC |
|  | (Probe5’-3')5'FAM-CTCCTGGATGAACTCCACCAGCAGA-3'BHQ1 |
|  |  |
| KC | (Forwardprimer5’-3’)GGCGCCTATCGCCAATG |
| (mouse) | (Reverseprimer5’-3')CTGGATGTTCTTGAGGTGAATCC |
|  | (Probe5’-3')5'FAM-CGCTGTCAGTGCCTGCAGACCATG-3'TAMRA |
|  |  |
| IFN-γ | (Forwardprimer5’-3’)TGGCATAGATGTGGAAGAAAAGAG |
| (mouse) | (Reverseprimer5’-3')TGCAGGATTTTCATGTCACCAT |
|  | (Probe5’-3')5'FAM-TTTTGCCAGTTCCTCCAGATATCCAAGAAGA-3'TAMRA |
|  |  |
| MIG | (Forwardprimer5’-3’)GAACCCTAGTGATAAGGAATGCA |
| (mouse) | (Reverseprimer5’-3')CTGTTTGAGGTCTTTGAGGGATT |
|  | (Probe5’-3')5'FAM-CATCAGCACCAGCCGAGGCACG-3'BHQ1 |
|  |  |
| IP-10 | (Forwardprimer5’-3’)AGTGCTGCCGTCATTTTCTG |
| (mouse) | (Reverseprimer5’-3')ATTCTCACTGGCCCGTCAT |
|  | (Probe5’-3')5'FAM-AGTCCCACTCAGACCCAGCAGG-3'BHQ1 |
|  |  |
| GADPH | (Forwardprimer5’-3’)CAACTACATGGTCTACATGTTC |
| (mouse) | (Reverseprimer5’-3')CTCGCTCCTGGAAGATG |
|  | (Probe5’-3')5'FAM-CGGCACAGTCAAGGCCGAGAATGGGAAGC-3'BHQ1 |
|  |  |
| TNF-α | (Forwardprimer5’-3’)GCACGATGCACCTGTACGAT |
| (Human) | (Reverseprimer5’-3')AGACATCACCAAGCTTTTTTGCT |
|  | (Probe5’-3')5'FAM-ACTGAACTGCACGCTCCGGGACTC-3′TAMRA |
|  |  |
| TRAIL | (Forwardprimer5’-3’)CTGAAGCAGATGCAGGACAAGTAC |
| (Human) | (Reverseprimer5’-3')GAAATGGTTTCCTCAGAGGTTCTCA |
|  | (Probe5’-3')FAM-AGCAGGGGCTGTTCATACTCTCTTCGT-BHQ1 |
|  |  |
| IL-6 | (Forwardprimer5’-3’)CAGCAACAATTCCTGGCGATA |
| (Human) | (Reverseprimer5’-3')AAGGCGAAAGCCCTCAATTT |
|  | (Probe5’-3')5'FAM-CTGCTGGCACCCAGCGACTCG-3′TAMRA |
|  |  |
| IL-8 | (Forwardprimer5’-3’)TCAGCTCTGCATCGTTTTGG |
| (Human) | (Reverseprimer5’-3')GTTCCATTATCCGCTACATCTGAA |
|  | (Probe5’-3')5'FAM-TTGGCTGTTACTGCCAGGACCCATATGT-3′TAMRA |
|  |  |
| MCP-1 | (Forwardprimer5’-3’) AATCATTCACCAGGCAAATTG |
| (Human) | (Reverseprimer5’-3') TTCTGTACTGCGGGTGGAAC |
|  | (Probe5’-3')5'FAM-TTCCTACCACCAGCAACCCTGCCA-3′TAMRA |
|  |  |
| IP-10 | (Forwardprimer5’-3’)GAAGATGTGCCTGTCCTGTGT |
| (Human) | (Reverseprimer5’-3')CGCTCAGGTCAGTGATGTTAA |
|  | (Probe5’-3')6Fam-TGGTGATGAGACCAGACTCCAGCTG-Tamra-p |
|  |  |
| GAPDH | (Forward primer 5’-3’)GAAGGTGAAGGTCGGAGTC |
| (Human) | (Reverse primer 5’-3')GAAGATGGTGATGGGATTTC |
|  | (Probe 5’-3')5′ FAM-CAAGCTTCCCGTTCTCAGCC-3′ TAMRA |
|  |  |
